# Knowledge, Attitudes, Risk Perception, Preparedness and Vaccine Intent of Health Care Providers towards the Nipah Virus in South India

**DOI:** 10.3390/tropicalmed7040056

**Published:** 2022-04-06

**Authors:** Lauren Himes, Veena Shetty, Sumathi Prabhu, Avinash K. Shetty

**Affiliations:** 1 Department of Pediatrics, Wake Forest School of Medicine, Medical Center Blvd, Winston-Salem, NC 27157, USA; lhimes@wakehealth.edu; 2 Department of Microbiology, K.S. Hegde Medical Academy, Nitte (Deemed to be University), Deralakatte, Mangaluru 575018, India; veenashetty@nitte.edu.in; 3 Department of Mathematics, Manipal Institute of Technology, Manipal 576104, India; k.sumathi@manipal.edu

**Keywords:** awareness, Deralakatte, Karnataka, Kerala, Nipah, physicians, nurses

## Abstract

Nipah virus (NiV) disease (NVD) remains a re-emerging public health threat in India. We assessed the knowledge, attitudes, and risk perception of NVD and future vaccine intent among a convenience sample of health care providers (HCP). The primary outcome measures were the knowledge, attitudes, and risk perception scores. Of 261 participants surveyed, 203 (77.8%) had heard of NiV and associated symptoms. The majority (248, 95%) identified the fruit bat as a primary NiV reservoir and 205 (79.8%) were aware of human-to-human transmission via droplets. Only 101 (38.7%) participants were aware that drinking date palm sap is a risk factor for transmission. Most HCP either agreed (117 (44.8%)) or strongly agreed (131 (50.2%)) that NiV is a serious illness. Less than half (121 (46.4%)) were aware of any institutional protocol for NiV; 235 (90.7%) of HCP stated that they need more information about prevention and treatment options. Knowledge scores were significantly higher among physicians compared to nurses whereas nurses and academic providers were more likely to have higher attitudes scores. A majority of respondents (20,779.9%) were willing to be vaccinated and willing to recommend the NiV vaccine to their patients (21,682.8%). Future strategies include education of HCP to bridge the knowledge gaps and enhance preparedness through disease-specific training for NiV infection.

## 1. Introduction

The Nipah virus (NiV) is a paramyxovirus that first appeared in Malaysia in 1998 [1]. Human NiV infection is most often characterized by fever, headache, dizziness, cough, and vomiting [2]. The clinical presentations may vary from subclinical infections to acute pulmonary infection and encephalitis [2,3]. Fruit bats (*Pteropus* bat species) represent the natural host of the NiV, but pigs were the intermediate host in the NiV outbreak in Malaysia [1]. From 2001–2014, a total of 33 outbreaks of NiV have been reported in both Bangladesh and India [4]. Transmission of NiV may occur following direct contact with infected animals, consumption of fresh date palm sap contaminated by bats, or from other infected individuals [1,5].

Nipah virus disease (NVD) remains a re-emerging public health threat in India, with outbreaks in 2018 associated with high mortality, followed by an outbreak in June 2019 and an isolated case in September 2021 [6,7,8,9]. Newspaper and media reports indicate that NiV-related stigma in the community and among health care providers (HCP) was a major barrier to providing appropriate care and support and re-integrate survivors [8]. Community concern is understandable considering that the case fatality rate in 2018 was 91%, with only two survivors [7]. Currently, there is still no known cure for NVD apart from supportive care [9].

In order to counteract the ongoing public health burden of NVD, there is a need for ongoing surveillance outbreak response [10]. The WHO research and development 2017 Blueprint included the NiV as a priority disease for rapid research and development; however, certain challenges unique to NiV make such education and awareness imperative [9,11]. For example, the range of symptoms associated with NVD is nonspecific [2]. Therefore, it is vital that HCP is prepared to recognize a wide array of presentations to minimize NVD transmission and to mitigate mortality.

In June 2019, the Government of Kerala published updated guidelines for diagnosing and treating NVD, but it is unclear whether surrounding regions have adequately updated their treatment protocols [12]. A study of NiV knowledge, attitudes, and practices found that medical interns at a teaching hospital in the city of Mangaluru demonstrated knowledge gaps and unsatisfactory practices about NiV [13]. In addition to responding to infectious disease outbreaks, HCP is also responsible for educating their patients. It is essential that HCPs accumulate the appropriate knowledge in order to best promote public health practices among community members and reduce the transmission of NiV.

The objective of the study was to assess NiV knowledge, attitudes, perception of risk, and preparedness (KAP) among HCPs and assess future vaccine intent. We hypothesized that HCPs in southern India are aware of NiV and its typical symptoms but would underestimate its public health burden. We also anticipated that HCPs would demonstrate stigmatized attitudes towards NiV infection and treatment, owing to its high infectivity and high mortality rate [7].

## 2. Materials and Methods

### 2.1. Study Design, Setting, and Population

This descriptive cross-sectional study surveyed a convenience sample of doctors, nurses, and paramedical staff (microbiologists and laboratory personnel). The study was conducted over a 6-week period from 6 June–20 July 2019, at K.S. Hedge Medical Academy (KSHEMA), a 1000-bed teaching hospital in Deralakatte, where over 1600 HCPs are employed [14]. Deralakatte is located in the south-eastern area of city of Mangaluru in the Dakshina Kannada district in the State of Karnataka, India. The Karnataka–Kerala State border is approximately 5.6 miles from Deralakatte.

The survey was based on prior studies investigating similar viral outbreaks [15,16,17]. The semi-structured anonymous questionnaire consisted of 7 sections (designated A–G). Section A identified demographic variables. Section B consisted of 20 knowledge statements to assess clinical presentation, modes of transmission, risk factors, and prevention of NiV. Responses were documented using True/False/I do not know responses. Section C contained eleven attitude statements. Section D contained four statements on risk perception, and Section E contained six preparedness statements to combat NiV. Sections C, D, and E had five answers choices on a Likert scale. Section E also contained three statements to assess the available treatment protocols. Section F (Sources of Information) contained 3 questions, and Section G (Vaccine Intent) measured views regarding NVD vaccine intent and experimental treatment. An expert panel of HCPs with expertise in infectious diseases, global health, public health, and virology reviewed the survey instrument. The questionnaire was administered in English, the commonly spoken language among HCP in our setting. The questionnaire was pilot tested among 30 random participants to determine the clarity and comprehension of the survey items.

Participants were selected using a convenience sampling method. Participants younger than 18 years of age and those not willing to participate were excluded. The survey participants were approached during the workday (Monday through Saturday), although some arranged for alternate interview times. Written informed consent was obtained from all participants. An interpreter who was fluent in both English and Kannada was available to clarify any questions with the consent process and survey material.

### 2.2. Scoring

Calculation of scale scores was performed using unweighted approach. Each accurate response to the 20 True/False knowledge statements was given one point, while zero points were awarded to an incorrect or “I do not know” or absent response. The knowledge scores obtained by the respondents ranged from 1 to 19 (i.e., 5% to 95%). Based on the distribution of the respondent scores, the cutoff score for possessing good knowledge was determined to be 14 questions correct (70% correct responses).

The attitude, risk, and preparedness were scored initially on a five-point scale, with 1, 2, and 3 indicating a negative attitude and fear regarding the virus and 4, 5 indicating a positive attitude or less fear regarding the virus. These five-point scores were then assigned 0 and 1 points each and categorized into two groups, both for attitude (negative/positive) and risk and preparedness (fear/less fear). A positive attitude score reflected a greater concern for the NiV, willingness to care for patients with NVD, and lack of fear and stigma towards patients with the disease. Based on the distribution of the scores of the respondents, the cutoff score for having a positive attitude was determined to be 8 (72.73%). The minimum score for risk perception was 1 and the maximum was 10. Based on the distribution of the respondent scores, a cutoff score of 8 (80%) was determined for the HCPs, showing their willingness to take risks and work with the infected patients.

The validity of the KAP questionnaire was confirmed by a Cronbach’s alpha internal consistency coefficient. It was 0.679 for Section B (virus knowledge and vaccine-related knowledge, which had only three options each) and 0.779 for Sections C, D, and E combined (attitude, risk perception, and preparedness, which had five options each).

### 2.3. Outcome Measures

Primary outcome measures were knowledge, attitude, and risk perception scores. These measures assessed the predictors of NiV outbreak preparedness among HCPs based on demographic characteristics. Secondary outcome measures included NiV vaccine intent and willingness to recommend the vaccine.

### 2.4. Statistical Analysis

All statistical analyses were carried out using the Statistical Package for the Social Sciences (version 20.0; SPSS, Chicago, IL, USA). The data were summarized using descriptive statistics. Knowledge, attitude, and preparedness scores were calculated. A *p*-value < 0.05 was considered statistically significant. After dichotomizing the data based on the cutoff scores, the groups were good knowledge vs. poor knowledge, positive attitude vs. negative attitude, and willingness vs. non-willingness. Thus, the categories were 14–20 (70–100%) vs. <14 (<70%), 08–11 (72.73–100%) vs. <08 (<72.73%) and 08–10 (80–100%) vs. <8 (<80%) for knowledge, attitude, and preparedness, respectively. Differences in NiV knowledge, attitude, and preparedness scores were examined by demographic characteristics using the chi-squared test for independence of attributes for categorical data such as gender, marital status, practice type, and location, and student’s t-test for two independent samples for continuous variables such as age and years of practice. Binary logistic regression analysis was performed to assess potential predictors of knowledge and attitude scores. Significant variables with *p* valve ≤ 0.05 were then entered in the binary logistic regression analysis model to identify which of these variables caused significant effect on the dependent variables (Knowledge score and Attitude score). The association measure was calculated using odds ratio and 95% CI. One-way analysis of variance (ANOVA) was performed to see whether the three groups of HCPs differed significantly in the KAP scores.

### 2.5. Ethical Consideration

The study protocol was approved by the Ethics Committee and the Institutional Review Boards at KSHEMA (8 April 2019) and Wake Forest School of Medicine (IRB00058292, 15 May 2019), respectively.

## 3. Results

### 3.1. Demographic Characteristics

Out of 288 consenting participants, 261 (90.6%) completed the 62-item structured questionnaire. Participants were excluded due to inconsistent or incomplete survey responses (>5% missing data in a section). Among the 261 participants, 96 (36.8%) were physicians, 71 (27.2%) were nurses, and 94 (36.0%) were allied health professionals. Over half of the participants were female (n = 170, 65%) and single (n = 134, 52%); one participant did not declare their marital status. The majority of the participants (n = 234, 90%) were employed at an academic center and practiced in an urban setting (n = 198, 76%); two participants did not specify their practice location. Based on the groups of the HCPs, the demographic characteristics of the study participants are summarized in Table 1.

### 3.2. Awareness and Sources of Information of Nipah Virus

Most respondents (n = 203; 77.8%) had heard of NiV and were aware of key symptoms. However, only n = 101 (38.7%) participants were aware that drinking date palm sap is a common risk factor for human infection. Most HCPs either agreed (n = 117; 44.8%) or strongly agreed (n = 131; 50.2%) that NiV is a serious illness. Less than half (n = 121; 46.4%) were aware of any protocol for NVD at their hospital site, and 235 (90.7%) of HCPs reported that they need more information about NVD prevention and treatment options. More than two-thirds of HCPs (n = 183; 70.1%) reported mass media as a primary information source. Only half of the respondents (n = 130; 49.8%) received information from expert health care professionals.

### 3.3. Knowledge Regarding Nipah Virus

Table 2 shows the responses of respondents to the knowledge of NiV transmission, risk factors, clinical presentation, and prevention. The mean knowledge score was 12.97 (64.87% correct responses) with a standard deviation 3.05 (15.26%). There were 122 (46.7%) respondents who had scores greater than or equal to 14 (70% correct responses). Table 3 shows the comparisons of demographic variables in relation to knowledge of NiV using chi-square test for independence of attributes. The demographic predictors of good knowledge scores include gender (*p* value 0.002) and type of practice (*p* value 0.011). The chi-square test for independence of attributes proved that the female respondents (n = 67; 54.9%) were more likely to have good knowledge about NiV than the males (n = 54; 44.3%). Moreover, HCPs in an academic setting (n = 116; 95.1%) had a better chance of having a good level of knowledge when compared to those involved in private practice (N = 6; 4.9%).

### 3.4. Attitudes towards Nipah Virus Infection

Table 4 shows the responses of the respondents to the attitude statements. The attitudes scores ranged from 0 to 11, with an overall mean of 8.91 (sd = 1.66). A total of 223 (85.4%) respondents had scores greater than or equal to 8 (72.73%), which was the cutoff for having a positive attitude. Table 5 gives the comparisons of demographic variables in relation to the attitude towards NiV using the chi-square test for independence of attributes. The demographic predictors of positive attitude scores include gender (*p* = 0.020) and type of practice (*p* = 0.007). The chi-square test for independence of attributes proved that the female respondents (n = 138; 61.9%) were more likely to have a positive attitude than males (n = 84; 37.7%). Moreover, providers in an academic setting (n = 206; 92.4%) had a better chance of having a positive attitude when compared to those in private practice (n = 17; 7.6%).

### 3.5. Risk Perception and Preparedness for Working with Patients with Nipah Virus Infection

Table 6 depicts the participant responses to the four statements on risk perception. Table 7 shows the responses of the respondents to the preparedness statements to combat NiV. The number of participants with positive preparedness scores (n = 106; 40.6%) was lower than that for having a positive attitude (n = 223; 85.4%). The majority (n = 203; 77.8%) of the participants knew of NiV, and 167 (64%) of HCP felt prepared to take care of patients with NiV. However, few HCPs (n = 33; 12.6%) stated that they have changed their practice to manage NiV risk, and less than half the study population (n = 121; 46.4%) were aware of any NiV protocol at their hospital. A majority of HCPs (n = 235; 90%) reported that they need more information about NiV prevention and treatment options. Table S1 gives the comparisons of demographic variables in relation to the risk perception and preparedness to work with people infected with NiV. There were no significant variables influencing the risk perception and preparedness scores.

### 3.6. Differences in Knowledge and Attitude Scores Based on Gender, Practice Type and Groups of Health Care Providers

Table 8 (and Table 3 and Table 5) shows that gender, type of practice, and the HCP groups cause a significant difference in knowledge and attitude scores. According to the binary logistic regression, only the HCP groups influenced the knowledge scores, whereas the HCP groups and the type of practice influenced the attitude scores. Based on logistic regression analysis (Table 9), nurses and HCPs working in academic settings significantly influenced the knowledge and attitude scores. Based on bivariate analysis using chi-squared test for independence of attributes, there are no significant demographic variables influencing the willingness to take risk in relation to working with patients with NiV infection (Table 10).

### 3.7. Vaccine Intent

If there was an approved NiV vaccine made available free of charge and recommended by the Government of India, 207 (79.9%) participants were willing to be vaccinated and 216 (82.8%) were willing to recommend it to their patients. None of the variables (demographic characteristics, knowledge and attitude scores, risk perception) were found to be significantly associated with vaccine intent or recommendations of the vaccine to others (Table 11).

## 4. Discussion

Many gaps exists regarding epidemiology of NiV [18]. Knowledge, attitudes, and preparedness measures have remained largely unexplored for NiV, especially in states outside of Kerala, India, where recent outbreaks have been reported [5,19]. Therefore, it is vital to survey surrounding regions, in order to determine whether HCPs are prepared to recognize and treat patients with NiV infection. Due to the high number of patients that travel daily from Kerala to Karnataka, it is likely that HCPs will encounter patients who have been exposed to NiV or have a confirmed infection.

All HCPs must have a robust knowledge of NiV transmission and infectivity to screen patients, as well as a well-informed index of suspicion for the associated symptoms. While most HCPs felt prepared to take care of patients with NVD, a small minority of providers stated that they have changed their practice to manage NVD risk, and less than half were aware of any protocol for NVD at their hospital site. Media sources (e.g., newspaper, television, and internet) were identified as their primary sources of information about NiV, and a strong majority of HCPs felt they need more information about NVD prevention and treatment options. Based on these results, it can be assumed that HCPs are willing to participate in educational programs, should they be made available. More training and education are warranted including appropriate triage and isolation precautions. While government guidelines had been released after the 2018 Kerala outbreak, our results indicate that hospitals outside the Kerala region had not effectively implemented the prescribed strategies [20].

Studies have found that sustained government support was instrumental in reframing the stakeholder response in Kerala. Initially, community members responded with fear-based hesitancy to institutional guidance regarding funeral procedures for individuals who had died of NVD. However, consistent communication between officials, community leaders, and stakeholders eventually led to broader acceptance of cremation instead of burial ceremonies. Ultimately, stakeholders were receptive to the “swift state response” that provided data-driven recommendations. Future efforts should appreciate the necessity of engaging community doubts and religious concerns prior to implementing government protocols [21].

Overall, physicians scored most highly on the knowledge section, followed by allied health professionals and nurses. Physicians may have a greater opportunity to enhance their knowledge compared with nurses and allied health professionals. Therefore, educational campaigns for different groups of HCPs should be targeted based on their specific needs, experience, and training.

Most HCPs either agreed or strongly agreed that NVD is a serious illness, and this attitude may impact patient care most notably. The majority of providers demonstrated positive attitude scores, but compared to physicians and allied health professionals, nurses were more willing to work with infected patients. Such initiative should not be underestimated given the stigma surrounding NVD and the increased risk for the acquisition of infection that HCPs face when caring for infected patients.

Working in an academic setting was another predictor of a better attitude score towards NiV. It is likely that the scope of practice of academic providers situates them to have a more positive outlook towards patients with NVD. In general, we would have expected a better attitude score to be associated with a higher knowledge score. However, we did not see a statistically significant difference in knowledge scores between academic and private practice physicians; this absence of difference may be due to the small number of private HCPs sampled in this study.

In our study, we did not find any attributes that seem to predispose an HCP to accept NiV or recommend NiV vaccine to their patients. Although a recombinant NiV vaccine has been in development since 2012, it has only recently entered a Phase 1 clinical trial [22,23]. Therefore, it is understandable that HCPs across all demographic variables may have limited knowledge and mixed attitudes toward the vaccine. Future studies should further explore the perceptions of the NiV vaccine as it continues to progress in the clinical trials. Furthermore, it is obvious that continued medical education for emerging and reemerging infectious diseases (such as NVD, COVID-19, etc.) should be prioritized so that HCPs are not burdened by the fear of uncertainty in the face of disease outbreaks.

This study is limited by a relatively small sample size of HCPs at a single site. Future studies should conduct similar analyses in other geographical regions, as well as within government hospitals, in order to generalize the study findings. We included paramedical staff in our study who do not treat patients as part of their job description. It is understandable that the preparedness scores for paramedical staff would be lower. We also did not specifically address the knowledge or attitudes related to personal protective equipment (PPE) or infection control protocols. Since PPE was not readily available and no formal hospital protocols were in place at the time of the study, we did not address either of these elements of NiV prevention. Finally, there is limited evidence that suggests that NiV may be transmitted through sexual intercourse. A recent report identified NiV in the semen of a NiV survivor, but this was published after our study was completed [24]. At this point, the correct answer to knowledge question #19 is ambiguous, but we still included it in the analysis, as there was no evidence to support sexual transmission at the time of the study.

## 5. Conclusions

Future strategies to bridge the knowledge gaps and enhance preparedness may be accomplished through continuous education efforts, hospital-wide protocol development, and disease-specific training for NiV. Given the gaps in knowledge regarding NiV between doctors and nurses, educational campaigns for different groups of HCPs should be targeted based on their specific needs, experience, and training. Further research on HCP levels of preparedness and awareness regarding NiV with a larger sample involving multiple sites should be conducted within the communities and government hospitals, as this information will promote a widespread commitment to education response and enhance the healthcare response in the event of an outbreak. A recent paper by Román et al. 2020 highlighted the key research needs for NiV preparedness, including animal surveillance, sound diagnostic practices, and dialogue between laboratory scientists and clinical practitioners that will enable enhanced technological advancement, as well as policy reform [25]. The global impact of the SARS-CoV-2 pandemic has urgently awoken international attention to the issue of Emerging Infectious Diseases, and universal buy-in for preparedness strategies is critical [25].

## Figures and Tables

**Table 1:** Demographic characteristics of study participants.

Demographic Variables		Groups of Health Care Providers (HCP)			Total = n	
		Physicians n = 96 (36.8%)	Nurses n = 71 (27.2%)	Allied Health Professionals n = 94 (36.0%)		Unanswered
Age in years	Mean ± sd	29.54 ± 12.08	28.87 ± 8.88	32.73 ± 9.47	n/a	n/a
	Range	21–89	20–57	21–71	n/a	
Gender	Males n (%)	42 (43.8)	4 (5.6)	45 (47.9)	91 (35%)	0
	Females n (%)	54 (56.3)	67 (94.4)	49 (52.1)	170 (65%)	
Marital Status	Married n (%)	36 (37.5)	35 (49.3)	55 (58.5)	126 (48%)	1
	Single n (%)	59 (61.5)	36 (50.7)	39 (41.5)	134 (52%)	
Type of practice	Private n (%)	2 (2.1)	10 (14.1)	15 (16)	27 (10%)	0
	Academic n (%)	94 (97.9)	61 (85.9)	79 (84)	234 (90%)	
Practice Location	Urban n (%)	71 (74)	51 (71.8)	76 (80.9)	198 (76%)	2
	Rural n (%)	23 (24)	20 (28.2)	18 (19.1)	61 (24%)	
Years of Practice	Mean ± sd	3.76 ± 6.65	5.21 ± 6.2	5.27 ± 6.65	n/a	n/a
	Range	0–40	0–35	0–40	n/a	

Note: sd denotes standard deviation.

**Table 2:** Knowledge regarding Nipah virus transmission, risk factors, clinical presentation, and prevention.

	Statement	Correct Answer	True (n/%)	False	I Don’t Know	Unanswered
1	Symptoms of Nipah virus infection may acute respiratory distress, convulsions, and coma	TRUE	231 (89.5)	11 (4.3)	16 (6.2)	3
2	A vaccine is currently available to prevent Nipah virus disease	FALSE	37 (14.5)	163 (63.7)	56 (21.9)	5
3	Fruit bats are the main reservoir of the Nipah virus	TRUE	248 (95.0)	7 (2.7)	6 (2.3)	0
4	The Nipah virus can be transmitted to humans from animals (such as bats or pigs)	TRUE	230 (88.8)	17 (6.6)	12 (4.6)	2
5	Human to human transmission of the Nipah virus has been reported in Bangladesh and India	TRUE	196 (75.4)	14 (5.4)	50 (19.2)	1
6	The Nipah virus was first discovered during the 2018 outbreak in Kerala State	FALSE	149 (57.1)	96 (36.8)	16 (6.1)	0
7	The Nipah virus can be transmitted from human to human via droplet infection	TRUE	205 (79.8)	33 (12.8)	19 (7.4)	4
8	Currently, there is no known treatment for the Nipah virus	TRUE	160 (62.5)	55 (21.5)	41 (16.0)	5
9	There are many strains of the Nipah virus	TRUE	110 (44.4)	17 (6.9)	121 (48.8)	13
10	Outbreaks of Nipah virus infection has occurred in Bangladesh and Malaysia	TRUE	164 (63.3)	20 (7.7)	75 (29.0)	2
11	Drinking date palm sap is a common risk factor for human Nipah virus infection	TRUE	101 (38.7)	46 (17.9)	110 (42.8)	4
12	Outbreaks of NiV occur most often during the summer months	FALSE	100 (39.2)	75 (29.4)	80 (31.4)	6
13	The Nipah virus can be spread through mosquitos	FALSE	9 (3.5)	218 (84.8)	30 (11.7)	4
14	It is possible to survive and recover from Nipah virus infection	TRUE	199 (78.3)	29 (11.4)	26 (10.2)	7
15	The Nipah virus does not cause disease in animals	FALSE	67 (26.0)	135 (52.3)	56 (21.7)	3
16	The 2018 Nipah virus outbreak in Kerala had a high mortality rate	TRUE	202 (78.0)	35 (13.5)	22 (8.5)	2
17	The Nipah virus can cause HIV/AIDS	FALSE	3 (1.2)	234 (90.0)	23 (8.8)	1
18	Nipah virus infection can be asymptomatic	TRUE	85 (33.5)	127 (50.0)	42 (16.5)	7
19	The Nipah virus can be passed on during sexual intercourse	FALSE	47 (18.1)	151 (58.1)	62 (23.8)	1
20	The Nipah virus can be cured with antibiotics	FALSE	31 (12.0)	183 (70.7)	45 (17.4)	2

**Table 3:** Comparison of the demographic variables in relation to the level of knowledge (chi-square test for independence of attributes).

Demographic Variables		Good knowledge n = 122 (46.7)	Poor Knowledge n = 139 (53.3)	Test Statistic	*p* Value
Age in years	Mean ± sd	31.3 ± 10.11	29.81 ± 10.71	1.151	0.251
Gender	Males n (%)	54 (44.3)	37 (26.6)	9.22	** 0.002
	Females n (%)	67 (54.9)	102 (73.4)		
Marital Status	Married n (%)	66 (54.1)	60 (43.2)	2.74	0.098
	Single n (%)	56 (45.9)	77 (55.4)		
Type of practice	Private n (%)	6 (4.9)	21 (15.1)	9.108	* 0.011
	Academic n (%)	116 (95.1)	118 (84.9)		
Practice Location	Urban n (%)	94 (77.1)	104 (74.8)	2.585	0.275
	Rural n (%)	27 (22.1)	34 (24.5)		
Years of Practice	Mean ± sd	4.89 ± 6.62	4.56 ± 6.49	0.398	0.691

* indicates significance; ** indicates high significance; sd indicates standard deviation; As noted in Table 1 and Table 2, if total n ≠ 261, then participants elected not to respond to the survey prompt.

**Table 4:** Attitude towards Nipah virus.

Statements		Strongly Disagree	Disagree	Uncertain/ Do Not Know	Agree	Strongly Agree	Unanswered
1	The Nipah virus may be prevented	5 (1.92)	10 (3.83)	29 (11.1)	151 (57.9)	66 (25.3)	0
2	The Nipah virus is a serious disease	4 (1.53)	4 (1.53)	5 (1.92)	117 (44.8)	131 (50.2)	0
3	I can help prevent the spread of the Nipah virus by educating my patients	2 (0.8)	3 (1.2)	7 (2.7)	133 (51.8)	112 (43.6)	4
4	The Nipah virus poses a serious public health threat in my country	3 (1.1)	20 (7.7)	21 (8.0)	125 (47.9)	92 (35.2)	0
5	I would be willing to care for a patient infected with the Nipah virus	2 (0.77)	14 (5.4)	33 (12.7)	141 (54.4)	69 (26.6)	2
6	Health care workers are at an increased risk of contracting the Nipah virus in the hospital setting	2 (0.78)	15 (5.8)	8 (3.1)	117 (45.5)	115 (44.8)	4
7	The media/internet/provides trustworthy information about Nipah	11 (4.3)	38 (14.9)	65 (25.5)	112 (43.9)	29 (11.4)	6
8	If someone in my family were to get Nipah, I would want it to remain private/secret	87 (33.3)	109 (41.8)	23 (8.8)	27 (10.3)	15 (5.7)	0
9	Patients with Nipah virus infection should not be stigmatized and discriminated against	32 (12.5)	19 (7.5)	23 (9.0)	94 (36.9)	87 (34.1)	6
10	The government provides trustworthy information about Nipah	5 (1.9)	17 (6.5)	41 (15.7)	156 (59.8)	42 (16.1)	0
11	Patients suffering from Nipah virus infection must be kept in isolation	2 (0.77)	5 (1.91)	13 (4.98)	120 (46.0)	121 (46.4)	0

**Table 5:** Comparison of the demographic variables in relation to the attitude (chi-square test for independence of attributes).

Demographic Variables		Positive Attitude n = 223 (85.4)	Negative Attitude n = 38 (14.6)	Test Statistic	*p* Value
Age in years	Mean ± sd	30.57 ± 10.54	30.13 ± 9.95	0.241	0.810
Gender	Males n (%)	84 (37.7)	7 (18.4)	5.38	* 0.020
	Females n (%)	138 (61.9)	31 (81.6)		
Marital Status	Married n (%)	110 (49.3)	16 (42.1)	0.763	0.382
	Single n (%)	111 (49.8)	22 (57.9)		
Type of practice	Private n (%)	17 (7.6)	10 (26.3)	9.884	** 0.007
	Academic n (%)	206 (92.4)	28 (73.7)		
Practice Location	Urban n (%)	167 (74.9)	31 (81.6)	1.179	0.555
	Rural n (%)	54 (24.2)	7 (18.4)		
Years of Practice	Mean ± sd	4.77 ± 6.53	4.4 ± 6.7	0.324	0.747

* indicates significance; ** indicates high significance; sd indicates standard deviation; As noted previously, if total n ≠ 261, then participants elected not to respond to the survey prompt.

**Table 6:** Risk Perception of Nipah virus infection.

		Strongly Disagree	Disagree	Uncertain/ Do Not Know	Agree	Strongly Agree	Unanswered
1	I accept the risk of contracting the Nipah virus as part of my job.	14 (5.4)	35 (13.5)	19 (7.3)	139 (53.5)	53 (20.4)	1
2	I have little control over whether or not I contract the Nipah virus.	25 (9.6)	51 (19.6)	55 (21.2)	108 (41.5)	21 (8.1)	1
3	I am afraid that I will contract the Nipah virus within the next year.	40 (15.3)	85 (42.6)	91 (34.9)	36 (13.8)	9 (3.4)	0
4	My colleagues are afraid that they will contract the Nipah virus.	29 (11.1)	64 (24.5)	88 (33.7)	66 (25.3)	14 (5.4)	0

**Table 7:** Preparedness for Working with Patients with Nipah virus infection.

		Strongly Disagree	Disagree	Uncertain/ Do Not Know	Agree	Strongly Agree	Unanswered
1	I feel prepared to take care of patients with Nipah virus infection	6 (2.3)	26 (10.1)	49 (19.1)	142 (55.3)	34 (13.2)	4
2	I feel prepared to recognize the symptoms and signs of Nipah virus infection and identify possible cases	4 (1.6)	21 (8.1)	41 (15.9)	162 (62.8)	30 (11.6)	3
3	I feel prepared to communicate the risk of acquiring the Nipah virus with my patients	4 (1.6)	18 (7.0)	31 (12.1)	170 (66.1)	34 (13.2)	4
4	People can take action to prevent contracting the Nipah virus in case of an outbreak in the country you live.	6 (2.3)	8 (3.1)	32 (12.4)	163 (63.1)	49 (19.0)	3
5	My institution is prepared to respond to an outbreak of the Nipah virus	1 (0.4)	18 (6.9)	59 (22.7)	135 (51.9)	47 (18.1)	1
6	Patients with a diagnosis of the Nipah virus infection must be admitted to a specialized Treatment Center	5 (1.9)	13 (5.0)	16 (6.2)	139 (53.5)	87 (33.5)	1

**Table 8:** Knowledge, attitudes, risk perception, and preparedness scores (in percentages) by groups of health care providers.

Demographic Variables		Groups of Health Care Providers (HCP)			Test Statistic	*p* Value
		Physicians n = 96 (36.8)	Nurses n = 71 (27.2)	Allied Health Professionals n = 94 (36.0)		
Knowledge Score	Mean ± sd	68.75 ± 15.66	57.68 ± 14.04	66.33 ± 13.97	12.421 £	** <0.001
	Range	25–95	5–85	30–95		
Proportion with good knowledge n (%)		60 (62.5)	15 (21.1)	47 (50)	28.69 €	** <0.001
Attitude Score	Mean ± sd	84.19 ± 10.90	76.44 ± 20.76	81.24 ± 12.84	5.577 £	** 0.004
	Range	45.45–100	0–100	45.45–100		
Proportion with positive attitude n (%)		90 (93.8)	53 (74.6)	80 (85.1)	11.985 €	** 0.002
Risk perception and preparedness score	Mean ± sd	64.89 ± 19.68	71.83 ± 19.22	63.61 ± 19.61	3.981 £	* 0.020
	Range	10–100	10–100	10–100		
Proportion with good risk perception and preparedness score		37 (38.5)	39 (54.9)	30 (31.9)	9.153 €	* 0.010

* indicates significance; ** indicates high significance; sd indicates standard deviation; As noted previously, if total number of respondents, n ≠ 261, then participants elected not to respond to that survey prompt. £ Test statistic values of one-way analysis of variance. Since the test statistic was highly significant, post hoc comparisons were done and Allied Health Professionals showed least significant difference among the three groups. € Test statistic values of chi-square test for independence of attributes.

**Table 9:** Predictors of good knowledge scores and attitude scores using binary logistic regression.

Dependent Variable	Predictors		Crude OR (95% CI)	Adjusted OR (95% CI)
Knowledge score	Groups	Physicians	1 (Reference)	1 (Reference)
		Nurses	3.73 (1.86, 7.52) *	3.70 (1.80, 7.63) *
		Allied Health Professionals	0.60 (0.34, 1.07)	0.74 (0.40, 1.35)
Attitude score	Groups	Physicians	1 (Reference)	1 (Reference)
		Nurses	1.94 (1.04, 4.24) *	2.53 (1.09, 5.85) *
		Allied Health Professionals	0.38 (0.14, 1.04)	0.49 (0.18, 1.40)
	Type of practice	Private	1 (Reference)	1 (Reference)
		Academic	4.31 (1.8, 10.33) *	4.03 (1.57, 10.42) *

CI, confidence interval; OR, Odds Ratio, * indicates significance.

**Table 10:** Bivariate analysis of demographic variables vs. willingness to take risk using chi-squared test for independence of attributes.

Demographic Variables		Willing to Take Risk n = 106 (40.6)	Not Willing to Take Risk n = 155 (59.4)	Test Statistic	*p* Value
Age in years	Mean ± sd	30.29 ± 11.41	30.66 ± 9.75	0.277	0.782
Gender	Males n (%)	36 (34)	55 (35.5)	0.085	0.771
	Females n (%)	70 (66)	99 (63.9)		
Marital Status	Married n (%)	49 (46.2)	77 (49.7)	0.421	0.516
	Single n (%)	57 (53.8)	76 (49.0)		
Type of practice	Private n (%)	14 (13.2)	13 (8.4)	2.55	0.279
	Academic n (%)	92 (86.8)	142 (91.6)		
Practice Location	Urban n (%)	79 (74.5)	119 (76.8)	0.393	0.821
	Rural n (%)	27 (25.5)	34 (21.9)		
Years of Practice	Mean ± sd	5.38 ± 7.71	4.26 ± 5.57	1.34	0.182

sd indicates standard deviation.

**Table 11:** Intention to receive a future NiV vaccine among Health Care Providers and Recommendation of vaccine to others. Statistical test used was chi-squared test for independence of attributes.

	Would be Willing to Accept NiV Vaccine				Would Recommend to Others			
Variables /Attributes	Subject in Analysis	N (%)	*p* Value	OR (95% Confidence Interval)	Subject in Analysis	N (%)	*p* Value	OR (95% Confidence Interval)
Gender								
Male	91	72 (79.1)	0.974	1.01 (0.54, 1.89)	91	77 (84.6)	0.548	0.81 (0.41, 1.61)
Female	169	134			169	138 (81.7)		
Age (in years)								
20–35	201	162 (80.6)	0.348	0.72 (0.37, 1.43)	201	166 (82.6)	0.893	1.05 (0.49, 2.28)
>35	60	45 (75)			60	50 (83.3)		
Marital Status								
Married	126	91 (72.2)	0.008 *	2.31 (1.24, 4.30)	126	100 (79.4)	0.178	1.56 (0.82, 2.99)
Single	133	114 (85.7)			133	114 (85.7)		
Specialization								
Physicians	96	79 (82.3)	0.622	1	96	85 (88.5)	0.163	1
Nurses	71	56 (78.9)		1.42 (0.7,2.89)	71	57 (80.3)		2.09 (0.94,4.64)
Allied Health Professionals	94	72 (76.6)		1.14 (0.54,2.4)	94	74 (78.7)		1.10 (0.51,2.37)
Years Practiced								
<5 years	188	155 (82.5)	0.045 *	0.53 (0.28, 0.99)	188	159 (84.6)	0.213	0.65 (0.33, 1.28)
> = 5 years	73	52 (71.2)			73	57 (78.1)		
Practice Type								
Private	27	23 (85.2)	0.448	0.65 (0.22,1.98)	27	24 (88.9)	0.395	0.59 (0.17,2.04)
Academic	233	184 (78.9)			233	192 (82.4)		
Practice Location								
Urban	199	159 (79.9)	0.674	0.86 (0.43,1.72)	199	164 (82.4)	0.791	1.11 (0.51,2.4)
Rural	62	48 (77.4)			62	52 (83.9)		
Knowledge Score								
Good/adequate knowledge (> = 14)	122	95 (77.9)	0.590	0.85 (0.47,1.55)	122	100 (81.9)	0.751	0.90 (0.47,1.71)
Poor knowledge (<14)	139	112 (80.6)			139	116 (83.5)		
Attitude score								
Positive attitude	223	174 (78.1)	0.215	0.54 (0.2,1.45)	223	184 (82.5)	0.798	0.89 (0.35,2.26)
Negative attitude	38	33 (86.8)			38	32 (84.2)		
Risk perception								
Willing to serve	106	87 (82.1)	0.362	1.34 (0.72,2.49)	106	91 (85.8)	0.274	1.46 (0.74,2.86)
Not willing to serve	155	120 (77.4)			155	125 (80.6)		

CI, confidence interval; NiV, Nipah virus; OR, Odds Ratio, * indicates significance; As noted previously, if total number of respondents, n ≠ 261, then participants elected not to respond to that survey prompt.

## Data Availability

The data presented in this study are available upon request from the corresponding author.

## Supplementary Materials

The following supporting information can be downloaded at: https://www.mdpi.com/article/10.3390/tropicalmed7040056/s1, Table S1: Comparison of the demographic variables in relation to the perception and practice.

## References

[1] Hauser N., Gushiken A.C., Narayanan S., Kottilil S., Chua J.V. (2021). Evolution of Nipah virus infection: Past, present, and future considerations. Trop. Med. Infect. Dis..

[2] World Health Organization (WHO) (2018). Nipah Virus.

[3] Kumar C.P.G., Sugunan A.P., Yadav P., Kurup K.K., Aarathee R., Manickam P., Bhatnagar T., Radhakrishnan C., Thomas B., Kumar A. (2019). Infections among Contacts of Patients with Nipah Virus, India. Emerg. Infect. Dis..

[4] Islam M.S., Sazzad H.M., Satter S.M., Sultana S., Hossain M.J., Hasan M., Rahman M., Campbell S., Cannon D.L., Ströher U. (2016). Nipah Virus Transmission from Bats to Humans Associated with Drinking Traditional Liquor Made from Date Palm Sap, Bangladesh, 2011–2014. Emerg. Infect. Dis..

[5] Sharma V., Kaushik S., Kumar R., Yadav J.P., Kaushik S. (2019). Emerging trends of Nipah Virus: A review. Rev. Med. Virol..

[6] Plowright R.K., Becker D.J., Crowley D.E., Washburne A.D., Huang T., Nameer P.O., Gurley E.S., Han B.A. (2019). Prioritizing Surveillance of Nipah Virus in India. PLoS Negl. Trop. Dis..

[7] Arunkumar G., Chandni R., Mourya D.T., Singh S.K., Sadanandan R., Sudan P., Bhargava B. (2019). Nipah Investigators People and Health Study Group. Outbreak Investigation of Nipah Virus Disease in Kerala, India, 2018. J. Infect. Dis..

[8] Spiropoulou C.F. (2019). Nipah Virus Outbreaks: Still Small but Extremely Lethal. J. Infect. Dis..

[9] World Health Organization (WHO) (2018). Nipah Virus—India.

[10] Chatterjee P. (2018). Nipah Virus Outbreak in India. Lancet.

[11] World Health Organization (WHO) (2017). 2017 Annual Review of Diseases Prioritized under the Research and Development Blueprint.

[12] Department of Health and Family Welfare (2019). NIPAH Virus Infection Guidelines for Surveillance, Diagnosis, Treatment, Prevention and Control. Government of Kerala. https://dhs.kerala.gov.in/wp-content/uploads/2021/09/Nipah-Guidelines-9-04-21-2-1.pdf.

[13] Pavithra H., Nirgude A.S., Balakrishna A.G., Bijali N., Revathi T.M., Yatnatti S.K. (2019). Are the medical interns ready to deal with the treatment, prevention and control of Nipah virus infection at the tertiary care hospital?. J. Fam. Med. Prim. Care.

[14] (2021). Anon. Justice K S Hegde Charitable Hospital. https://kshegdehospital.in/.

[15] Jalloh M.F., Robinson S.J., Corker J., Li W., Irwin K., Barry A.M., Ntuba P.N., Diallo A.A., Jalloh M.B., Nyuma J. (2017). Knowledge, Attitudes, and Practices Related to Ebola Virus Disease at the End of a National Epidemic—Guinea, August 2015. MMWR Morb. Mortal. Wkly. Rep..

[16] Wong L.P., AbuBakar S. (2013). Health Beliefs and Practices Related to Dengue Fever: A Focus Group Study. PLoS Negl. Trop. Dis..

[17] Rolison J.J., Hanoch Y. (2015). Knowledge and Risk Perceptions of the Ebola virus in the United States. Prev. Med. Rep..

[18] Ray A., Mittal A. (2020). Nipah Virus Infection: Gaps in Evidence and its Public Health Importance. Public Health.

[19] Pallivalappil B., Ali A., Thulaseedharan N.K., Karadan U., Chellenton J., Dipu K.P., Anoop Kumar A.S., Sajeeth Kumar K.G., Rajagopal T.P., Suraj K.P. (2020). Dissecting an Outbreak: A Clinico-epidemiological Study of Nipah Virus Infection in Kerala, India, 2018. J. Glob. Infect. Dis..

[20] Nipah Advisory Group (2019). Directorate of Health Services, Kerala: Nipah Virus Infection Guidelines.

[21] Chandrasekharan P.K., Rahul A., Gopakumar R.N.S., Nair A.T.S. (2020). Stakeholder Perspective of Handling the Deceased during the Nipah Virus Outbreak in Kerala, South India, 2018. Am. J. Trop. Med. Hyg..

[22] Auro Vaccines LLC (2020). A Phase 1 Randomized, Placebo-controlled, Observer-blind Trial to Assess the Safety and Immunogenicity of a Nipah Vaccine, HeV-sG-V (Hendra Virus Soluble Glycoprotein Vaccine), in Healthy Adults. NCT04199169 Auro Vaccines LLC. NCT04199169.

[23] Geisbert T.W., Bobb K., Borisevich V., Geisbert J.B., Agans K.N., Cross R.W., Prasad A.N., Fenton K.A., Yu H., Fouts T.R. (2021). A single dose investigational subunit vaccine for human use against Nipah virus and Hendra virus. NPJ Vaccines.

[24] Arunkumar G., Abdulmajeed J., Devadiga S., Sushama A., Sam R., Jayaram A., Radhakrishnan C., Sajeeth K.K.G., Sakeena K., Vasudevan J. (2019). Persistence of Nipah virus RNA in semen of survivor. Clin. Infect. Dis..

[25] Gómez Román R., Wang L.-F., Lee B., Halpin K., de Wit E., Broder C.C., Rahman M., Kristiansen P., Saville M. (2020). Nipah@20: Lessons Learned from Another Virus with Pandemic Potential. mSphere.

