# Supplementary material for: Knowledge, Attitudes, Risk Perception, Preparedness and Vaccine Intent of Health Care Providers towards the Nipah Virus in South India
Source: Trop Med Infect Dis. 2022 Apr 6;7(4):56. doi: 10.3390/tropicalmed7040056 (PMC9031456; doi:10.3390/tropicalmed7040056)
Supplement: Supplementary file 1 [file tropicalmed-07-00056-s001.zip › tropicalmed-1579445-supplementary.pdf]

Table S1. Comparison of the demographic variables in relation to the perception & practice.

Demographic Variables		Willing to Take Risk n = 106 (40.6)	Not Willing to Take Risk n = 155 (59.4)	Test Statistic	p Value
Age in years	Mean ± sd	30.29±11.41	30.66±9.75	0.277	0.782
Gender	Males n (%)	36 (34)	55 (35.5)	0.085	0.771
	Females n (%)	70 (66)	99 (63.9)		
Marital Status	Married n (%)	49 (46.2)	77 (49.7)	0.421	0.516
	Single n (%)	57 (53.8)	76 (49.0)		
Type of practice	Private n (%)	14 (13.2)	13 (8.4)	2.55	0.279
	Academic n (%)	92 (86.8)	142 (91.6)		
Practice Location	Urban n (%)	79 (74.5)	119 (76.8)	0.393	0.821
	Rural n (%)	27 (25.5)	34 (21.9)		
Years of Practice	Mean ± sd	5.38 ± 7.71	4.26 ± 5.57	1.34	0.182

* indicates significance ** indicates highly significance sd indicates standard deviation.
